# Clinical value of urinary cytokines/chemokines as prognostic markers in patients with crescentic glomerulonephritis

**DOI:** 10.1038/s41598-022-13261-7

**Published:** 2022-06-17

**Authors:** Junseok Jeon, Jeeeun Park, Hyo Jin Boo, Kyeong Eun Yang, Cheol-Jung Lee, Jung Eun Lee, Kyunga Kim, Ghee Young Kwon, Wooseong Huh, Dae Joong Kim, Yoon-Goo Kim, Hye Ryoun Jang

**Affiliations:** 1 Division of Nephrology, Department of Medicine, Samsung Medical Center, Sungkyunkwan University School of Medicine, Seoul, Republic of Korea; 2 Division of Scientific Instrumentation & Management, Korea Basic Science Institute, Daejeon, Republic of Korea; 3 Statistics and Data Center, Research Institute for Future Medicine, Samsung Medical Center, Seoul, Republic of Korea; 4 Department of Pathology, Samsung Medical Center, Sungkyunkwan University School of Medicine, Seoul, Republic of Korea

**Keywords:** Kidney diseases, Chemokines, Cytokines

## Abstract

Crescentic glomerulonephritis (CrGN) usually requires urgent immunosuppressive treatment. However, aggressive immunosuppressive treatment is often difficult because of the patients’ medical conditions or comorbidities. Prognostic markers including urinary cytokines/chemokines as noninvasive biomarkers were explored in CrGN patients. This prospective cohort study included 82 patients with biopsy-confirmed CrGN from 2002 to 2015 who were followed up for 5 years. Urine and serum cytokines/chemokines on the day of kidney biopsy were analyzed in 36 patients. The median age was 65 years and 47.6% were male. Baseline estimated glomerular filtration rate (eGFR) and interstitial fibrosis and tubular atrophy (IFTA) scores were identified as significant prognostic factors. Among patients with cytokines/chemokines measurement, increased IL-10 level was identified as an independent predictor of good prognosis, and increased levels of urinary MCP-1 and fractalkine tended to be associated with good prognosis after adjusting for baseline eGFR and IFTA score. However, semiquantitative analysis of intrarenal leukocytes did not show prognostic value predicting renal outcome or correlation with urinary cytokines/chemokines. This study supports the clinical importance of baseline eGFR and IFTA scores and suggests potential usefulness of urinary IL-10, MCP-1, and fractalkine as prognostic markers for predicting renal outcomes in patients with CrGN.

## Introduction

Crescentic glomerulonephritis (CrGN) is often fatal and can progress to end-stage kidney disease. Timely initiation of immunosuppressive treatment following urgent kidney biopsy has been regarded as standard management^1^. However, some patients develop end-stage kidney disease despite immunosuppressive treatment, and therapy-related complications can seriously impair quality of life and even lead to death, especially in the elderly or patients with serious comorbidities^2, 3^. Therefore, the risk–benefit profile and potential treatment response should be considered before initiating aggressive immunosuppressive treatment. Previous studies have reported baseline kidney function or pathologic findings at diagnosis as prognostic markers predicting the treatment response of CrGN^4–6^. However, predicting renal outcomes in CrGN using noninvasive biomarkers remains challenging.

Crescent formation, a main feature of pauci-immune GN, anti-glomerular basement membrane (GBM) GN, and aggressive immune complex GN, can be triggered by various underlying disorders. Glomerular microvascular injury and rupture of GBM result in leakage of plasma proteins and inflammatory cells within the Bowman’s space, and subsequent hyperplasia of parietal epithelial cells even in non-inflammatory glomerular injury^7, 8^. The cellular composition of crescents appears to change over time, with predominant epithelial cells in the early stages and increasing numbers of infiltrating macrophages, lymphocytes, and myofibroblasts in later stages^9, 10^. Eventually, complex inflammatory processes are involved in the development and evolution of crescents^11, 12^.

Several studies have investigated the roles of cytokines or chemokines, such as monocyte chemoattractant protein-1 (MCP-1)^13, 14^, regulated upon activation in normal T cells expressed and secreted (RANTES)^14, 15^, interleukin (IL)-6^16^, IL-10^17^, and transforming growth factor-β1 (TGF-ß1)^18^ in the pathogenesis of CrGN. However, few studies have attempted to determine the clinical value of cytokines/chemokines in urine or serum specimens of patients with CrGN, focusing on prognosis or treatment response, and the clinical relevance of urinary or serum cytokines/chemokines is yet to be determined in CrGN^18–21^.

In this study, we aimed to investigate the clinical value of urinary cytokines/chemokines as biomarkers for predicting the prognosis of CrGN.

## Results

### Baseline characteristics

The baseline characteristics of patients with CrGN are presented in Table 1. The median age at diagnosis was 65 years, and 39 (47.6%) patients were men. Baseline eGFR was 18.3 (median, IQR 10.0–27.9) mL/min/1.73 m^2^ and uPCR was 1.97 (median, IQR 1.18–3.67) mg/mgCr. Baseline eGFR was significantly higher in the good prognosis group than in the poor prognosis group. Approximately 70% of the patients showed positive results for anti-neutrophil cytoplasmic antibody (ANCA). Pauci-immune crescentic glomerulonephritis was the most common pathologic diagnosis, followed by immune complex-mediated crescentic glomerulonephritis. Patients with underlying autoimmune disease other than ANCA-associated diseases or who were treated with immunosuppressive therapy prior to the diagnosis of CrGN were not included. The definitions of good and poor prognosis were specified in the "Clinical data and primary outcome" subsection of the "Material and methods" section.

**Table 1:** Baseline characteristics and outcomes of all patients with crescentic glomerulonephritis.

Variables	Total patients (n = 82)	Poor prognosis (n = 36)	Good prognosis (n = 46)	*P*
Age, years	65 (51–72)	66 (50–73)	64 (51–71)	0.85
Male, n (%)	39 (48)	17 (47)	22 (47)	0.96
WBC, 10^3^/µL	8.61 (6.92–11.20)	8.58 (7.16–10.31)	8.65 (6.74–12.16)	0.56
CRP, mg/dL	2.39 (0.6–7.1)	1.12 (0.2–8.2)	4.15 (0.8–7.3)	0.08
eGFR, mL/min/1.73 m^2^	18.3 (10.0–27.9)	10.7 (7.9–23.7)	20.2 (12.7–35.1)	0.002
uPCR, mg/mgCr	1.97 (1.18–3.67)	2.17 (0.92–5.3)	1.91 (1.28–2.59)	0.85
ANCA positive, n (%)	58 (71)	26 (72)	32 (70)	0.79
Anti-GBM positive, n (%)	1	1	0	1.00
**Pathologic diagnosis, n (%)**				0.73
Pauci-immune GN	60 (73.2)	25 (69.4)	35 (76.1)	
Immune complex-mediated GN	12 (14.6)	5 (13.9)	7 (15.2)	
IgA nephropathy	2 (2.4)	1 (2.8)	1 (2.2)	
Membranous GN	1 (1.2)	1 (2.8)	0	
Mesangioproliferative GN	1 (1.2)	1 (2.8)	0	
Crescentic GN, unspecified^a^	6 (7.3)	3 (8.3)	3 (6.5)	
Dialysis requirement at diagnosis	19 (23.2)	12 (33.3)	7 (15.2)	0.10
**Histological findings**				
Normal glomeruli, %	30 (16–51)	21 (8–37)	43 (24–54)	0.003
Active crescent, %	25 (9–46)	25 (6–49)	25 (10–43)	0.80
Glomerular chronic change, %	31 (18–59)	44 (22–65)	25 (15–46)	0.01
Tubular chronic change, n (%)				0.01
IFTA score 0	13 (16)	2 (6)	11 (24)	
IFTA score 1	40 (49)	16 (44)	24 (52)	
IFTA score 2	15 (18)	10 (28)	5 (11)	
IFTA score 3	11 (13)	8 (22)	3 (7)	
**Immunosuppressive treatment**^**b**^**, n (%)**				0.09
None	5 (6)	4 (11)	1 (2)	
Treatment 1	7 (9)	4 (11)	3 (7)	
Treatment 2	57 (70)	20 (56)	37 (80)	
Treatment 3	13 (16)	8 (22)	5 (11)	
Death, n (%)	11 (13.4)	8 (22.2)	3 (6.5)	0.08
Kidney transplantation, n (%)	6 (7.3)	3 (8.3)	3 (6.5)	1.00
End-stage kidney disease^c^, n (%)	27 (32.9)	21 (58.3)	6 (13.0)	< 0.001

Variables are shown as n (%) or median (interquartile range).

ANCA, antineutrophil cytoplasmic antibody; CRP, C-reactive protein; eGFR, estimated glomerular filtration rate; GN, glomerulonephritis, IFTA, interstitial fibrosis and tubular atrophy; uPCR, urine protein to creatinine ratio; WBC, white blood cells.

^a^No immunofluorescence or electron microscopy was performed adequately for a specific diagnosis due to insufficient biopsy tissue.

^b^Treatment 1, 2, and 3: steroid alone, steroids with other immunosuppressants, and steroids with other immunosuppressants and plasmapheresis, respectively.

^c^End-stage kidney disease was defined as requirement of maintenance dialysis or kidney transplantation.

### Clinical and histological data

**Figure 1:**
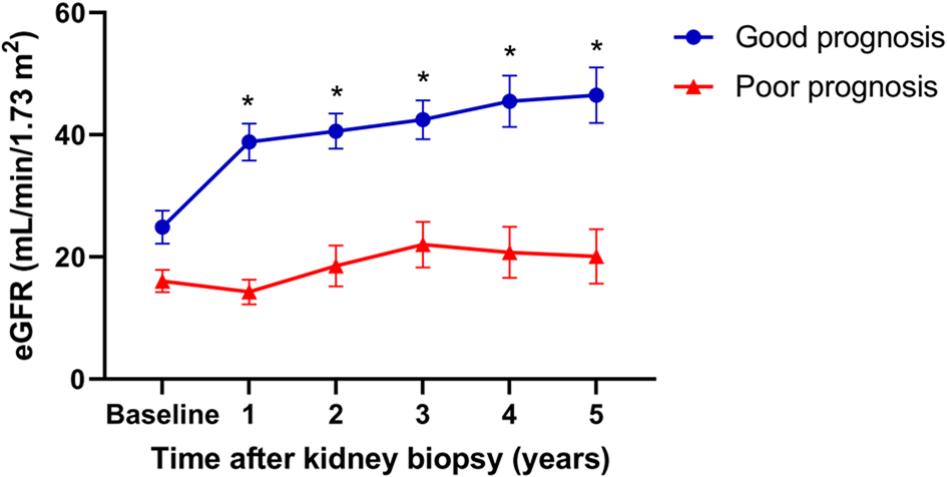
Mean estimated glomerular filtration rate (eGFR) changes for five consecutive years from the time of kidney biopsy. Renal function between 1 and 5 years after kidney biopsy maintained stably in the good prognosis group. Data are expressed as mean ± standard error of mean. Group comparisons of each time point were performed using mixed-effects analysis. *P < 0.05 versus poor prognosis group at each time point after Šidák corrections for multiple comparisons.

Among the variables reflecting histologic features, percentages of normal glomeruli, glomerular chronic changes, and tubular chronic changes were significantly different according to prognosis. There was no significant difference in the treatment regimens, and 60 (86%) patients were treated with corticosteroids and other immunosuppressants (mostly cyclophosphamide). There was no difference in the characteristics between the cytokine measurement and non-measurement groups (Supplemental Table 1). When comparing eGFR and uPCR data from 1 to 5 years after diagnosis, renal function remained stable in the good prognosis group (Fig. 1).

### Association of clinical variables and histological findings with prognosis

Table 2 presents the results of univariable and multivariable logistic regression analyses for good prognosis. In the univariable logistic regression, ln(C-reactive protein), ln(eGFR), and the proportions of normal glomeruli, glomerular chronic changes, and tubular chronic changes were associated with prognosis. Multivariable logistic analysis using variables that were significant at P < 0.1 in the univariable analyses, showed a significant association of ln(eGFR) with good prognosis (odds ratio [OR], 2.74; 95% confidence interval [Cl], 1.01–7.43, *P* = 0.048). Tubular chronic changes, IFTA 2 and 3, tended to be associated with poor prognosis (IFTA 2: OR, 0.15; 95% Cl 0.02–1.23; *P* = 0.08; IFTA 3: OR, 0.11; 95% Cl 0.01–1.00; *P* = 0.05).

**Table 2:** Predictors of good prognosis in all patients with crescentic glomerulonephritis.

Variables	Univariable		Multivariable	
	OR (95% CI)	*P*	OR (95% CI)	*P*
Age	1.01 (0.98–0.04)	0.66		
Male	1.03 (0.43–2.45)	0.96		
ln(CRP)	1.33 (1.00–1.76)	0.047	1.34 (0.94–1.90)	0.11
ln(eGFR)	3.01 (1.43–6.33)	0.004	2.74 (1.01–7.43)	0.048
ln(uPCR)	0.83 (0.28–2.45)	0.73		
Vasculitis serology marker	0.88 (0.34–2.30)	0.79		
**Histologic features**				
Normal glomeruli (%)	1.03 (1.01–1.05)	0.01	0.99 (0.97–1.03)	0.85
Active crescents (%)	1.00 (0.98–1.02)	0.81		
Glomerular chronic change (%)	0.98 (0.96–0.99)	0.01	0.99 (0.96–1.01)	0.35
Tubular chronic change		0.02		
IFTA 1 (versus IFTA 0)	0.27 (0.05–1.40)	0.12	0.24 (0.04–1.46)	0.12
IFTA 2 (versus IFTA 0)	0.09 (0.01–0.58)	0.01	0.15 (0.02–1.23)	0.08
IFTA 3 (versus IFTA 0)	0.07 (0.01–0.51)	0.009	0.11 (0.01–1.00)	0.05
**Immunosuppressive treatment (reference: none)**^**a**^		0.11		
Treatment 1	3.00 (0.21–42.63)	0.42		
Treatment 2	7.40 (0.77–70.77)	0.08		
Treatment 3	2.50 (0.21–29.26)	0.47		
CD 45 positive cells (%)	0.99 (0.89–1.09)	0.78		
CD 3 positive cells (%)	1.02 (0.93–1.11)	0.73		
CD 20 positive cells (%)	0.98 (0.88–1.09)	0.71		

Logistic regression analyses were performed to determine the prognosis. Multivariable analyses were performed using variables with P < 0.1 in univariable analysis.

CI, confidence interval; CRP, C-reactive protein; eGFR, estimated glomerular filtration rate; IFTA, interstitial fibrosis and tubular atrophy; ref, reference; OR, odds ratio; uPCR, urine protein to creatinine ratio.

^a^Treatment 1, 2, and 3: steroid alone, steroids with other immunosuppressants, and steroids with other immunosuppressants and plasmapheresis, respectively.

### Urinary cytokines/chemokines associated with prognosis

**Figure 2:**
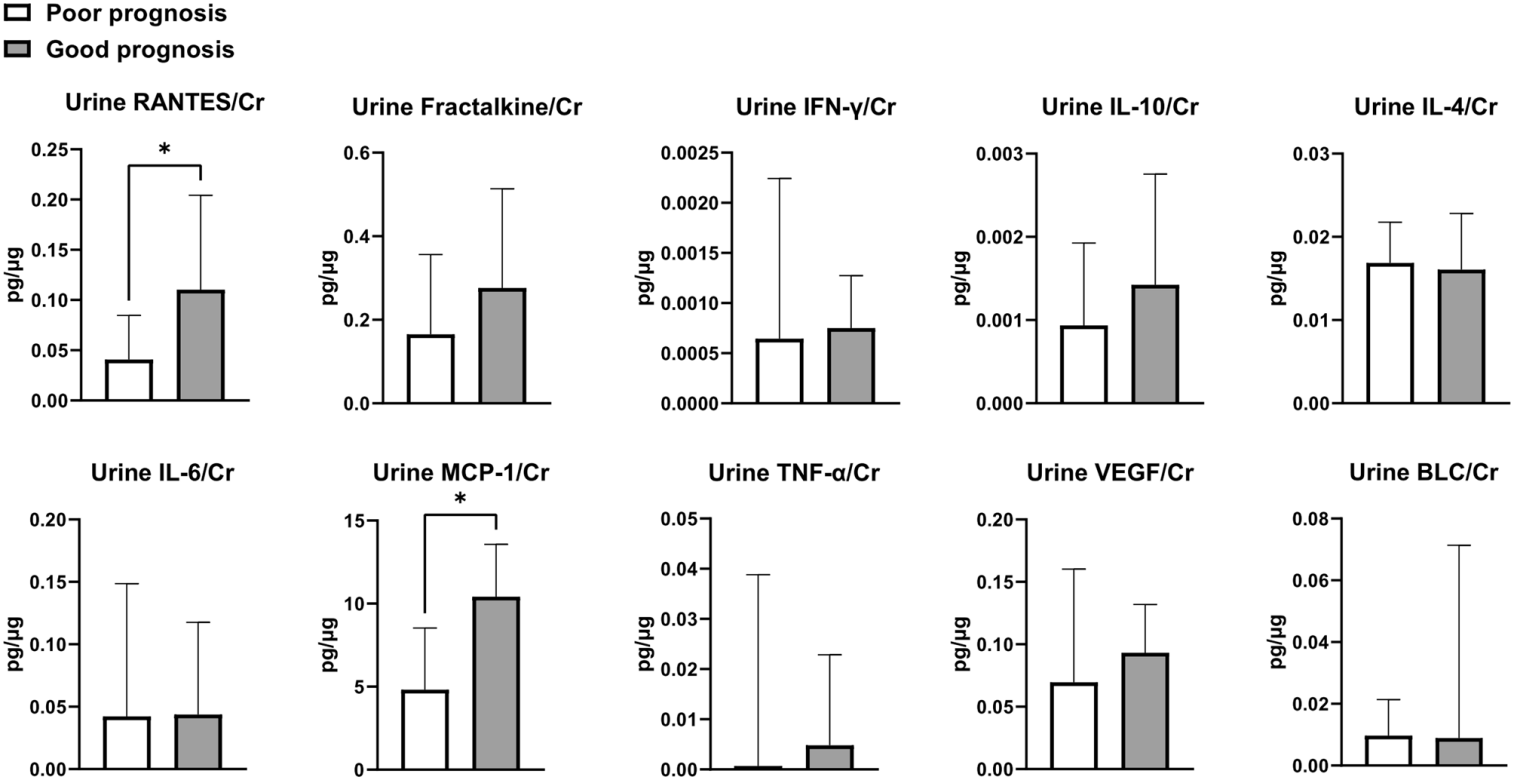
Comparison of urine cytokines/chemokines levels between good prognosis and poor prognosis groups. Data are expressed as median ± interquartile range. *P < 0.05. The Mann–Whitney test was used for comparison. BLC, B lymphocyte chemokine; Cr, creatinine; IFN, interferon; IFTA, interstitial fibrosis tubular atrophy; IL, interleukin; RANTES, regulated on activation, normal T cell expressed and secreted; TNF, tumor necrosis factor; VEGF, vascular endothelial growth factor.

Ten cytokines/chemokines were measured in the blood and urine specimens of 36 patients. Table 3 shows serum cytokines/chemokines, and Fig. 2 shows urinary cytokines/chemokines according to prognosis. Baseline urine RANTES/creatinine ratio and urine MCP-1/creatinine ratio were higher in the good prognosis group than in the poor prognosis group. In univariable analysis, ln(urine RANTES/creatinine) (OR, 1.99; 95% Cl 0.97–4.05; *P* = 0.06), ln(urine IL-10/creatinine) (OR, 2.13; 95% Cl 0.91–4.99; *P* = 0.08), and ln(urine MCP-1/creatinine) (OR, 2.18; 95% Cl 0.96–4.95; *P* = 0.06) tended to be associated with good prognosis after treatment (Table 4). Multivariable analysis for each cytokine/chemokine was performed with ln(eGFR) and tubular chronic change (IFTA score). After adjusting ln(eGFR) and tubular chronic change, ln(urine IL-10/creatinine) (OR, 2.50; 95% Cl 1.01–6.21; *P* = 0.048) was associated with a good prognosis. Ln(urine fractalkine/creatinine) (OR, 2.94; 95% Cl 0.91–9.44; *P* = 0.070) and ln(urine MCP-1/creatinine) (OR, 2.27; 95% Cl 0.90–5.72; *P* = 0.081) tended to be associated with good prognosis.

**Table 3:** Serum cytokines/chemokines (n = 36).

Cytokine/chemokines, median [IQR]	Total	Poor	Good
Serum RANTES, pg/mL	117,300 (59,515–201,368)	97,290 (38,634–185,167)	141,428 (67,840–209,952)
Serum Fractalkine, pg/mL	77.2 (4.2–130.6)	87.4 (0–230)	67.5 (16.7–116.5)
Serum IFN-γ, pg/mL	6.9 (1.5–17.6)	7.4 (1.4–12.7)	4.5 (1.7–25.2)
Serum IL-10, pg/mL	0.07 (0–17.90)	0 (0–19.5)	2.71 (0–18.9)
Serum IL-4, pg/mL	0 (0–0)	0 (0–0)	0 (0–3.76)
Serum IL-6, pg/mL	2.8 (0–43.3)	1.1 (0–59.3)	10.1 (0.9–40.3)
Serum MCP-1, pg/mL	626 (324–852)	571 (271–872)	632 (376–850)
Serum TNF-α, pg/mL	37 (19–65)	35 (18–74)	37 (19–53)
Serum VEGF, pg/mL	284 (127–564)	220 (105–865)	330 (141–547)
Serum BLC, pg/mL	162 (97–256)	104 (68–227)	162 (106–294)

Variables are shown as median (interquartile range).

BLC, B lymphocyte chemokine; IFN, interferon; IFTA, interstitial fibrosis tubular atrophy; IL, interleukin; IQR, interquartile range; MCP, monocyte chemoattractant protein; RANTES, regulated on activation, normal T cell expressed and secreted; TNF, tumor necrosis factor; VEGF, vascular endothelial growth factor.

**Table 4:** Predictors of good prognosis in the cytokines/chemokines measurement group (N = 36).

Urine cytokines/chemokines	Unadjusted		Adjusted^a^	
	OR (95% CI)	*P*	OR (95% CI)	*P*
ln(urine RANTES/creatinine)	1.99 (0.98–4.05)	0.06	1.94 (0.88–4.28)	0.10
ln(urine fractalkine/creatinine)	2.01 (0.77–5.24)	0.15	2.94 (0.91–9.44)	0.07
ln(urine IFN-γ/creatinine)	0.95 (0.46–2.00)	0.90	0.94 (0.39–2.28)	0.94
ln(urine IL-10/creatinine)	2.13 (0.91–4.99)	0.08	2.50 (1.01–6.21)	0.048
ln(urine IL-4/creatinine)	1.56 (0.56–4.32)	0.39	1.84 (0.60–5.68)	0.29
ln(urine IL-6/creatinine)	1.23 (0.75–2.02)	0.41	1.50 (0.79–2.84)	0.21
ln(urine MCP-1/creatinine)	2.18 (0.96–4.95)	0.06	2.27 (0.90–5.72)	0.08
ln(urine TNF-α/creatinine)	1.23 (0.91–1.66)	0.17	1.27 (0.93–1.76)	0.14
ln(urine VEGF/creatinine)	1.06 (0.46–2.46)	0.89	0.97 (0.39–2.38)	0.94
ln(urine BLC/creatinine)	1.20 (0.76–1.89)	0.44	1.47 (0.82–2.62)	0.20

BLC, B lymphocyte chemokine; CI, confidence interval; IFN, interferon; IFTA, interstitial fibrosis tubular atrophy; IL, interleukin; MCP, monocyte chemoattractant protein; OR, odds ratio; RANTES, regulated on activation, normal T cell expressed and secreted; TNF, tumor necrosis factor; VEGF, vascular endothelial growth factor.

^a^Adjusted for ln(eGFR) and tubular chronic change (IFTA score).

### Analyses of intrarenal immune cells and urinary cytokines/chemokines

**Figure 3:**
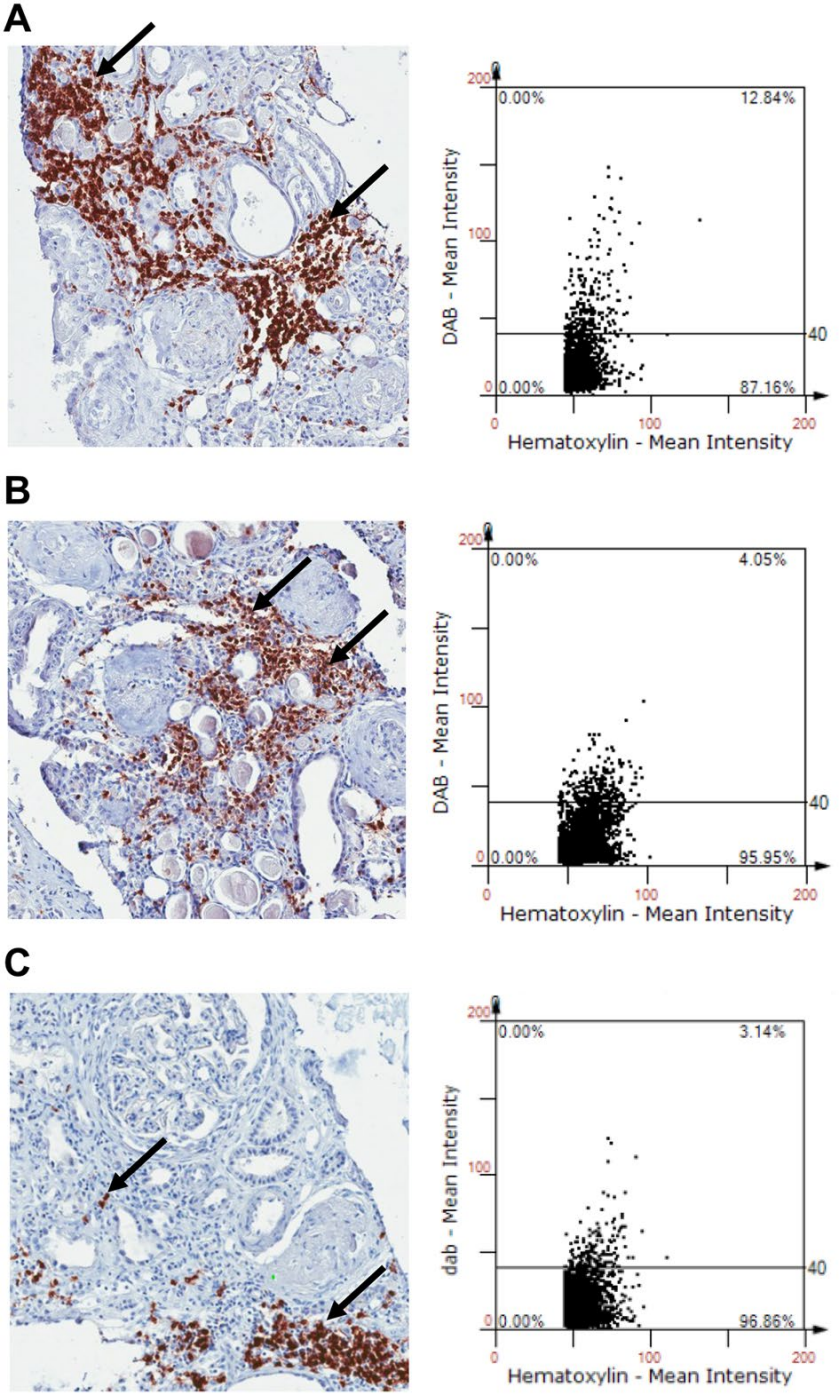
Semiquantitative analysis of intrarenal leukocytes infiltration in biopsy tissue of crescentic glomerulonephritis. Representative images of tissueFAXS analysis for immunohistochemical staining with anti-cluster of differentiation (CD) 45, anti-CD3, and anti-CD20 antibodies. (**A**) CD 45, (**B**) CD 3, (**C**) CD 20. The arrows indicate anti-CD45, CD3, or CD20 positive cells.

Immunohistochemistry of CD45, CD3, and CD20 followed by tissueFAXS analysis was performed in 78 patients whose kidney biopsy specimens were available (Fig. 3). The infiltration of total leukocytes expressing CD45, T cells expressing CD3, and B cells expressing CD20 was comparable in the good prognosis and poor prognosis groups [good prognosis vs. poor prognosis: CD45, 9.2% (4.5–13.8) vs. 8.3% (5.1–16.7), *P* = 0.81; CD3, 8.0% (3.7–10.3) vs. 7.0% (3.7–10.2), *P* = 0.69, CD20, 2.4% (0.5–4.7) vs. 1.1% (0.5–4.2), *P* = 0.57]. The degree of infiltration of intrarenal immune cells did not show any correlation with urinary cytokines/chemokines or histologic features, such as chronic glomerular changes (CD45, r = -0.058, *P* = 0.62; CD3, r = -0.045, *P* = 0.70; CD20, r = -0.104, *P* = 0.36) and tubular chronic changes [IFTA score 0 vs. 1 vs. 2 vs. 3: CD 45 9.3% (6.6–1.36) vs. 9.1% (4.0–13.4) vs. 6.0% (4.4–11.0) vs. 12.4% (4.8–18.0), *P* = 0.63; CD3 6.1% (3.5–8.6) vs. 7.4% (3.5–9.8) vs. 7.0% (4.6–9.6) vs. 10.3% (6.7–12.0), *P* = 0.44; CD20, 1.3% (0.6–3.6) vs. 3.1% (0.3–5.7) vs. 1.6% (0.5–3.7) vs. 1.7% (0.8–2.8), *P* = 0.91].

## Discussion

In this study, we investigated clinical variables, histological findings, serum and urine cytokines/chemokines levels, and intrarenal leukocyte infiltration to identify prognostic factors predicting the prognosis of CrGN. Baseline low eGFR and extensive chronic tubular change (IFTA 2 or 3) were identified as independent predictors of poor prognosis. Our study demonstrated that high baseline levels of urinary IL-10, MCP-1, and fractalkine were predictors of good prognosis independent of baseline eGFR and chronic tubular change.

Previous studies have reported baseline renal function and renal histopathological findings at the time of diagnosis as predictors of renal outcome in CrGN^5, 6, 22^. In 2010, Berden et al.^4^ incorporated the results of previous studies and proposed histopathologic classification of ANCA-associated glomerulonephritis based on glomerular pathology assessed by light microscopy: focal, crescentic, mixed, and sclerotic; although later validation studies have shown conflicting results regarding crescentic and mixed classes^23^. In addition to glomerular findings, tubulointerstitial histopathological findings have also been reported as predictors of renal outcome^6, 24^. In our study, consistent with previous studies, baseline kidney function and tubulointerstitial histopathological findings at diagnosis were important predictors of renal outcome.

In our study, a high urinary IL-10 level was identified as a significant prognostic factor for good renal outcome, independent of baseline eGFR and IFTA score. IL-10, a representative anti-inflammatory cytokine, is released by regulatory T cells and is associated with immune regulation in CrGN^25^. IL-10–deficient regulatory T cells lose their capacity to attenuate renal tissue injury in CrGN^17^ and low intracellular IL-10 levels in remission were associated with a high relapse rate of ANCA-associated vasculitis during the long-term follow-up^26^. Therefore, the increase in urinary IL-10 at diagnosis may suggest endogenous initiation of immune modulation switching the intrarenal immunologic micromilieu in a favorable direction in which disease will be easily controlled by showing enhanced responsiveness to immunosuppressive treatment.

High levels of urinary MCP-1 and fractalkine also tended to be associated with good prognosis in our study, although not statistically significant, probably owing to the small sample size. Urinary RANTES was high in the good prognosis group, although it was not a significant predictor in the multivariable logistic regression analysis. MCP-1, fractalkine, and RANTES attract T lymphocytes and macrophages. MCP-1 and fractalkine play prominent roles in the recruitment of macrophages, whereas RANTES plays a more significant role in the recruitment of T lymphocytes^27^. Blocking MCP-1 and fractalkine attenuated kidney damage in a murine model of GN^14, 28^. Cellular crescents can be reversed by appropriate immunosuppressive treatment in the early stages^14, 29^. Interstitial collagen production and progression to fibrous crescents are common when the Bowman’s capsule ruptures, accompanied by an influx of fibroblasts and macrophages into the Bowman’s space^30^. Macrophages play a central role in the formation of glomerular crescents by stimulating tissue factor expression and fibrin deposition, and subsequently promote the chronic fibrotic phase in established CrGN^31^. Ablation of macrophages attenuated kidney injury with reduced glomerular crescents, and adoptive transfer of macrophages aggravated murine crescentic glomerulonephritis^32, 33^. These previous reports suggest that factors affecting the recruitment or activity of macrophages may be important in the pathogenesis of CrGN^30–33^. Both MCP-1 and RANTES were reported to play important roles in the inflammatory phase of CrGN, and blocking of MCP-1 reduced the number of crescents and deposition of type I collagen, whereas blocking of RANTES did not^14^. In another study, urinary MCP-1 was higher in patients with active renal vasculitis than in those with inactive renal vasculitis and correlated with glomerular macrophage infiltration, and decreased urinary MCP-1 following treatment preceded the improvement in renal function^19^. Although further studies are needed to confirm our findings, urine MCP-1 may be used as a potential predictor of good prognosis, and urine fractalkine or RANTES may also be considered as candidates for noninvasive biomarkers in CrGN.

In this study, infiltration of total leukocytes, T cells, and B cells in the kidney tissue of CrGN patients was analyzed semiquantitatively. Studies analyzing leukocyte infiltration in the kidney tissue of CrGN patients through immunohistochemistry showed predominant interstitial T lymphocyte infiltration and predominant intraglomerular monocyte or macrophage infiltration^34, 35^. One study showed that intrarenal infiltration of helper T cells and macrophages was correlated with response to methylprednisolone therapy in patients with rapidly progressive GN, except for anti-GBM disease^36^, and another study reported that intraglomerular total leukocytes infiltration as well as interstitial or intraglomerular macrophage infiltration in patients with ANCA-associated GN was correlated with serum creatinine at the time of biopsy, but not after 1 year^35^. Although semiquantitative analysis of intrarenal total leukocytes, T cells, and B cells did not predict renal outcome in our study, these results may support the potential importance of urinary biomarkers reflecting the entire intrarenal immunologic micromilieu because it is difficult and time consuming to analyze each immune cell in kidney biopsy tissue.

Our study has several limitations. First, despite the prospective design of this study, blood and urine samples were collected from only 36 of the 82 patients. However, the strength of our study is that serum and urine specimens were simultaneously collected on the day of kidney biopsy. Moreover, as there were no differences in clinical characteristics between the cytokine/chemokine measurement and the non-measurement groups, we believe that the cytokine measurement group can represent all study subjects. Second, changes in cytokines/chemokines after biopsy were not evaluated. Some studies reported that changes following initial treatment or during the post-treatment follow-up period were associated with prognosis^19, 20^. However, our study design may have increased relevant in real-world practice as urinary cytokines/chemokines at the time of diagnosis are highly significant in determining immunosuppressive treatment. Third, we included all CrGN patients with various etiologies, although ANCA-associated pauci-immune GN was the main cause of CrGN. Although each subtype of CrGN has a different pathophysiology, CrGN usually requires aggressive immunosuppressive treatment regardless of the underlying etiology. In addition, common processes of disruption of GBM integrity, development of glomerular crescent, and progression to fibrosis and sclerosis are involved in the pathophysiology of CrGN^37^. Therefore, it is reasonable to assume that common biomarkers can be used to predict overall prognosis including the treatment response in CrGN.

Despite these limitations, our study has some strengths. We reviewed five consecutive years of laboratory data to analyze renal outcomes, and it is a relatively sufficient follow-up period compared with previous studies for prognostic biomarkers. We investigated diverse factors to identify predictors of therapeutic response and prognosis of CrGN, including measurements of serum and urine cytokines/chemokines and immunohistochemistry of biopsy specimens.

In conclusion, high urinary levels of IL-10, MCP-1, and fractalkine may be used as potential noninvasive prognostic markers to determine immunosuppressive treatment for CrGN. Further prospective studies with a large cohort are required to validate the clinical usefulness of these biomarkers in CrGN.

## Material and methods

### Study design and patient selection

In a prospective cohort of all patients undergoing kidney biopsy between 2002 and 2015 at the Samsung Medical Center (Seoul, South Korea), patients with biopsy-proven “CrGN” were analyzed. We excluded patients younger than 18 years, with CrGN of transplanted kidney, and without available data of 1-year spot urine protein to creatinine ratio or any evidence of clinical response from electronic medical records. A total of 82 CrGN cases were included in the final analysis.

The study was approved by the Institutional Review Board of Samsung Medical Center in compliance with the Declaration of Helsinki (IRB numbers: 2010-03-007 and 201707099), and informed written consent was obtained from all participants.

### Clinical data and primary outcome

Clinical data including age, sex, prescription history of immunosuppressants, history of plasmapheresis or hemodialysis, and events necessary for clinical response assessment, such as the beginning of maintenance dialysis or kidney transplantation, were extracted from electronic medical records. Laboratory data, including estimated glomerular filtration rate (eGFR), spot urine protein to creatinine ratio (uPCR), and spot urine albumin to creatinine ratio on the day of kidney biopsy (baseline) and at 1 year after kidney biopsy, were collected. Changes in eGFR up to 5 years after kidney biopsy were analyzed to evaluate long-term renal outcomes. Serum creatinine-based eGFR was calculated using the Modification of Diet in Renal Disease equation^38^. Baseline total white blood cell counts, erythrocyte sedimentation rate, and C-reactive protein levels were also collected. In addition, titers of anti-proteinase 3 antibody, anti-myeloperoxidase antibody, and ANCA were analyzed. Positive ANCA results without elevation of anti-proteinase 3 and anti-myeloperoxidase antibody levels were considered false positives. Treatment regimens were categorized into steroid alone (treatment 1), steroids with other immunosuppressants (treatment 2), and steroids with other immunosuppressants and plasmapheresis (treatment 3).

The study outcome was divided into good and poor prognosis groups. A good prognosis was defined as a 1-year uPCR decrement of > 50% and increased or maintained eGFR. The increase in uPCR, decrease in eGFR, maintenance dialysis or kidney transplantation within 1 year, or death within 1 year after initial kidney biopsy were considered a poor prognosis.

### Analysis of histological data

Biopsy reports of all patients with CrGN were thoroughly reviewed. The percentages of global sclerosis and each type (cellular, fibrocellular, and fibrous) of crescent were scored by a pathologist in a blinded manner. Then, the proportions of normal glomeruli (without crescents or global sclerosis), glomeruli with active crescents (cellular or fibrocellular crescents), and glomeruli with chronic changes (fibrous crescents or global sclerosis) were analyzed. The tubular chronic changes were organized according to the interstitial fibrosis and tubular atrophy (IFTA) scoring system as follows: 0, absent; 1, mild (< 25%); 2, moderate (25–50%); and 3, severe (> 50%) of the total tubulointerstitial area^39^.

### Immunohistochemistry and TissueFAXS analysis

We performed immunohistochemical staining for cluster of differentiation (CD) 45 (leukocyte common antigen), CD3 (surface marker of T cells), and CD20 (transmembrane protein of B cell surface) using formalin-fixed renal tissue sections, as described previously^40^. Renal tissue sections (4-μm thick) were deparaffinized using xylene, rehydrated in a graded alcohol series, and placed in a citrate buffer solution (pH 6.0). The slides were placed in a pressure cooker and heated with microwaves for 10 min to enhance antigen retrieval. After cooling, the slides were immersed in hydrogen peroxide solution (Dako, Carpinteria, CA) for 30 min to block endogenous peroxidase. After overnight incubation with serum-free protein block (Dako) at 4 °C, the slides were incubated at 25 °C for 1 h with a 1:100 dilution of monoclonal rat anti-mouse antibody against CD45, CD3, and CD20 (BD Biosciences, San Jose, CA). Next, they were incubated at room temperature for 30 min with a mixed solution of dextran coupled with peroxidase molecules and goat secondary antibody molecules (Dako). Then, 3,3′-diaminobenzidine tetrahydrochloride (Dako) was applied to the slides to show a brown color, followed by counterstaining with Mayer’s hematoxylin (Dako). A TissueFAXS workstation (Tissue Gnostics, Vienna, Austria) was used to analyze and calculate the percentages of CD45-, CD3-, and CD20-positive cells infiltrated into renal tissues, as described previously^40^.

### Measurements of cytokines/chemokines

Urine and serum samples were collected from 36 patients on the day of the kidney biopsy. Several cytokines/chemokines, including RANTES, fractalkine, interferon-gamma, IL-10, IL-4, IL-6, MCP-1, tumor necrosis factor-alpha, and vascular endothelial growth factor were measured using the MILLIPLEX^®^ MAP Human Cytokine/Chemokine panel (Merck Millipore Corp., Billerica, MA), coupled with a Luminex xMAP^®^ platform in a magnetic bead format. These cytokines/chemokines were measured according to the manufacturer's instructions as described in our previous study^41^. B lymphocyte chemoattractant was measured using a human CXCL13/BLC/BCA-1 Quantikine ELISA kit (R&D Systems, Minneapolis, MN) according to the manufacturer's instructions. Urine cytokine/chemokine levels were adjusted for urine creatinine levels. The measured values of cytokine/chemokine were logarithmically transformed for statistical analysis owing to skewed distribution.

### Statistical analysis

Demographics and baseline clinical data are presented as median (interquartile range [IQR]) or number (percentage), as appropriate. We compared the variables of the cytokine measurement group and the non-measurement group using Pearson’s Chi-square test or Fisher’s exact test and Student’s *t*-test to confirm that the cytokine measurement group represented all patients in our study. For immunohistochemistry and cytokine/chemokine data, the Mann–Whitney U-test was performed to analyze the differences according to prognosis. Spearman’s correlation analysis was performed to investigate the associations of immunohistochemistry and cytokines/chemokines with prognosis and other clinical variables. To evaluate the prognostic value of cytokines/chemokines and clinical variables in predicting patient outcomes, univariable and multivariable logistic regression analyses were used. All statistical analyses were performed using SAS version 9.4 (SAS Institute Inc., Cary, NC). Statistical significance was defined as a two-sided *P*-value < 0.05.

## Supplementary Information

Supplementary Table 1.

## Data Availability

The datasets used and/or analyzed during the study are available from the corresponding author upon reasonable request. The data are not publicly available due to privacy/ethical restrictions.

## Abbreviations

ANCA: Anti-neutrophil cytoplasmic antibody
CrGN: Crescentic glomerulonephritis
eGFR: Estimated glomerular filtration rate
GBM: Glomerular basement membrane
GN: Glomerulonephritis
IFTA: Interstitial fibrosis and tubular atrophy
IL: Interleukin
MCP-1: Monocyte chemoattractant protein-1
RANTES: Regulated upon activation in normal T cells expressed and secreted
uPCR: Urine protein to creatinine ratio
