# Supplementary material for: Clinical value of urinary cytokines/chemokines as prognostic markers in patients with crescentic glomerulonephritis
Source: Sci Rep. 2022 Jun 17;12:10221. doi: 10.1038/s41598-022-13261-7 (PMC9205991; doi:10.1038/s41598-022-13261-7)
Supplement: Supplementary file 1 — Supplementary Table 1. [file 41598_2022_13261_MOESM1_ESM.pdf]

Supplemental Table 1. Baseline characteristics according to the measurements of urinary and serum cytokines/chemokines

Variables	Non-measurement (N=46)	Measurement (N=36)	*P*
Age, years	66 (57-73)	60 (49-70)	0.14
Male, n (%)	22 (48)	17 (47)	1.00
WBC, 10^3/μL	8.62 (6.92-12.89)	8.60 (7.31-11.11)	0.97
CRP, mg/dL	2.3 (0.8-6.5)	2.5 (0.4-7.8)	0.55
eGFR, mL/min/1.73 m^2	18.4 (9.3-28.0)	16.5 (10.6-27.5)	0.80
uPCR, mg/mgCr	1.9 (1.1-4.4)	2.2 (1.3-3.0)	0.60
ANCA positive, n (%)	34 (74)	24 (67)	0.64
Histological findings	18.4 (9.3-28.0)	16.5 (10.6-27.5)	0.80
Normal glomeruli	31 (15-50)	23 (18-55)	0.95
Cellular or fibrocellular crescent, %	28 (10-50)	23 (4.5-39)	0.11
Glomerular chronic change, %	26 (20-46)	43 (14-67)	0.36
Tubular chronic change, n (%)			0.16
IFTA score 0	5 (11)	8 (23)	
IFTA score 1	25 (57)	15 (43)	
IFTA score 2	6 (14)	9 (26)	
IFTA score 3	8 (18)	3 (9)	
Immunosuppressive treatment [a], n (%)			0.25
None	2 (4)	3 (8)	
Treatment 1	5 (11)	2 (6)	
Treatment 2	29 (63)	28 (78)	
Treatment 3	10 (22)	3 (8)	
Good prognosis	24 (52)	22 (61)	0.56

Variables are shown as numbers (%) or medians (interquartile ranges).

[a]Treatment 1, 2, and 3: steroid alone, steroids with other immunosuppressants, and steroids with other immunosuppressants and plasmapheresis, respectively.

ANCA, antineutrophil cytoplasmic antibody; CRP, C-reactive protein; eGFR, estimated glomerular filtration rate; IFTA, interstitial fibrosis and tubular atrophy; uPCR, urine protein to creatinine ratio; WBC, white blood cells
